# Explosive growth of facet joint interventions in the medicare population in the United States: a comparative evaluation of 1997, 2002, and 2006 data

**DOI:** 10.1186/1472-6963-10-84

**Published:** 2010-03-30

**Authors:** Laxmaiah Manchikanti, Vidyasagar Pampati, Vijay Singh, Mark V Boswell, Howard S Smith, Joshua A Hirsch

**Affiliations:** 1 Pain Management Center of Paducah, 2831 Lone Oak Road, Paducah, KY, 42003, USA; 2 Pain Diagnostics Associates, 1601 Roosevelt Road, Niagara WI 54151, USA; 3 Texas Tech. University Health Sciences Center, 3601 4th St MS: 8182 Lubbock TX 79430, USA; 4 Albany Medical College, 47 New Scotland Avenue, Albany NY 12208, USA; 5 Massachusetts General Hospital, 55 Blossom St., Gray 289, Boston, MA 02114, USA

## Abstract

**Background:**

The Office of Inspector General of the Department of Health and Human Services (OIG-DHHS) issued a report which showed explosive growth and also raised questions of lack of medical necessity and/or indications for facet joint injection services in 2006.

The purpose of the study was to determine trends of frequency and cost of facet joint interventions in managing spinal pain.

**Methods:**

This analysis was performed to determine trends of frequency and cost of facet joint

Interventions in managing spinal pain, utilizing the annual 5% national sample of the Centers for

Medicare and Medicaid Services (CMS) for 1997, 2002, and 2006.

Outcome measures included overall characteristics of Medicare beneficiaries receiving facet joint interventions, utilization of facet joint interventions by place of service, by specialty, reimbursement characteristics, and other variables.

**Results:**

From 1997 to 2006, the number of patients receiving facet joint interventions per 100,000

Medicare population increased 386%, facet joint visits increased 446%, and facet joint interventions increased 543%. The increases were higher in patients aged less than 65 years compared to those 65 or older with patients increasing 504% vs. 355%, visits increasing 587% vs. 404%, and services increasing 683% vs. 498%.

Total expenditures for facet joint interventions in the Medicare population increased from over $229 million in 2002 to over $511 million in 2006, with an overall increase of 123%. In 2006, there was a 26.8-fold difference in utilization of facet joint intervention services in Florida compared to the state with the lowest utilization - Hawaii.

There was an annual increase of 277.3% in the utilization of facet joint interventions by general physicians, whereas a 99.5% annual increase was seen for nurse practitioners (NPs) and certified registered nurse anesthetists (CRNAs) from 2002 to 2006. Further, in Florida, 47% of facet joint interventions were performed by general physicians.

**Conclusions:**

The reported explosive growth of facet joint interventions in managing spinal pain in certain regions and by certain specialties may result in increased regulations and scrutiny with reduced access.

## Background

The Office of Inspector General (OIG)of the Department of Health and Human Services (OIG-DHHS), issued a report in September 2008 [1] noting that Medicare paid over $2 billion in 2006 for interventional pain management (IPM) procedures. This report also showed that Medicare payments for facet joint injections increased from $141 million in 2003 to $307 million in 2006. Of concern, 63% of facet joint injection services allowed by Medicare in 2006 did not meet the Centers for Medicare and Medicaid Services (CMS) program requirements, resulting in approximately $129 million in improper payments. This report illustrated that facet joint injection services provided in an office were more likely to have an error than those provided in an ambulatory surgery center (ASC) or hospital outpatient department (HOPD). The OIG report also illustrated that approximately 35% of the Medicare facet joint injections were performed by non-interventional pain physicians. The OIG report recommended some radical changes in monitoring utilization of interventional techniques. Further, independent investigators also have shown an exponential increase in the performance of facet joint interventions [2-5].

Friedly et al [3,6] reviewed trends in injection procedures focusing mainly on epidural injections from 1994 to 2001. Manchikanti et al [2] analyzed the growth of all interventional techniques in managing chronic pain in Medicare beneficiaries from 1997 to 2006. Both investigators demonstrated an overall increase of interventional techniques in all settings and in all parts of the country. The increase in the number of patients receiving IPM services per 100,000 of Medicare recipients was 137% with an overall increase of IPM services of 197% per 100,000 Medicare beneficiaries increasing by 197%. However, the most dramatic increase was found to be for facet joint interventions with a 543% increase per 100,000 Medicare beneficiaries.

Chronic spinal pain in the United States is highly prevalent with substantial economic impact [7-16]. However, the treatment of spinal pain is controversial, in part related to the wide variability in the treatments utilized [16]. The rising prevalence of chronic low back pain has been demonstrated with continued high levels of disability and health care use [7]. Freburger et al [7] showed an annual increase of 11.6% of chronic low back pain and attributed a substantial portion of rising low back pain care costs over the past 2 decades to this rising prevalence. Chronic spinal pain is associated with functional and psychological disabilities and health, social, and economic impact, especially in the elderly [10-13,17,18].

Epidural injections and facet joint interventions are the 2 most commonly utilized procedures in IPM [1-6,19,20]. However, the literature addressing the effectiveness of facet joint interventions, though emerging, is highly variable, based on the technique, outcome measures, patient selection, and methodology [21-28].

Health care spending in the United States is escalating and the long-range fiscal sustainability of Medicare is in question [26-29]. In a report titled *Accounting for the Cost of US Health Care: A New Look at Why Americans Spend More *[30] it was found that in 2006 the United States spent $650 billion more on health care than any of its peer Organisation for Economic Co-operation and Development (OECD) countries, even after adjusting for wealth. The majority of the excess spending was derived from outpatient care. One of the means of controlling health care expenditures is by ensuring that all care is medically necessary and avoiding overuse, abuse, and fraud. The OIG report suggests that there is significant overuse, abuse, and potential fraud in performing facet joint interventions in the United States.

In this study, we sought to evaluate the use of all types of facet joint interventions (i.e., intraarticular injections, facet joint nerve blocks, and facet joint neurotomy) in the lumbar, cervical, and thoracic spine. In addition, our purpose was to identify trends in the number of procedures, reimbursement, specialty involvement, fluoroscopy use, and indications. Finally, we sought to explore the association between overall injection costs and the volume of services provided in HOPD settings, ACSs, and in-office settings.

## Methods

The data for this study was used from the standard 5% national sample of the CMS physician outpatient billing claims for 1997, 2002, and 2006. The data set is a sample of those enrolled in the fee-for-service Medicare program based on selecting records with specific numbers in positions 8 and 9 of the health insurance claim number and is generated by CMS. The CMS 5% sample data set is therefore unbiased and unpredictable in terms of any patient characteristics, but does allow appropriate tracking of patients over time and across databases. Consequently, CMS makes this 5% sample available to researchers. In addition, a 100% data set is so large that it is not feasible to use for research purposes. Thus, Institutional Review Board (IRB) approval was not required. CMS's providing the data also does not require IRB approval prior to analysis or publication.

Previous studies [3,6] generally included patients aged 65 and older. We have studied all patients enrolled in Medicare who received interventional techniques [2]. Overall Medicare enrolled over 43 million beneficiaries in 2006, and is the single largest health care payor in the United States [31]. Consequently, the Medicare data set includes a large proportion of procedures for spinal pain being performed in the United States, including facet joint interventions. In addition to patient age, the database included the Current Procedural Terminology (CPT) procedure codes; the International Classification of Diseases, 9^th ^Revision, Clinical Modification (ICD-9-CM) diagnosis codes; date of service, provider specialty, provider zip code, and allowed charges.

To yield data for the entire beneficiary population of Medicare, results from the 5% sample were multiplied by 20. In addition, rates were calculated based on Medicare beneficiaries for the corresponding year and are reported as per 100,000 Medicare beneficiaries. The data were tabulated based on the place of service - HOPD, ASC, or office for the years 1997, 2002, and 2006. Facility charges were also identified for HOPDs, ASCs, and offices (office facility portion as overhead expense equals total office payment minus physician payment). Facility payments for HOPD were estimated based on national payment rates with consideration of modifiers, due to the non-availability of HOPD data in the data set. Allowed charges were used to estimate the costs of Medicare for these procedures and costs were adjusted for health care inflation using the U.S. Bureau of Labor Statistics Consumer Price Index (CPI) for medical care services and represent costs for 2006 [32].

In this study, all types of facet joint interventions with CPT codes 64470, 64472, 64475, 64476, 64622, 64623, 64626, and 64627, with evaluation of Medicare data of 1997, 2002, and 2006 were utilized. Appropriate considerations were given to the changes in the CPT with introduction of new codes or replacement codes.

In addition, diagnostic codes were utilized from the ICD-9-CM. The previous studies excluded cervical and thoracic facet joint interventions [3,6]; they argued that cervical and thoracic spine disorders differ clinically from lumbar spine disorders and may be the result of different disease processes. They believed that cervical and thoracic interventions represent a very small proportion of patients. However, the emerging statistics show that cervical and thoracic facet joint interventions occupy a large proportion of facet joint interventions. Thus, it was felt essential to include these interventions.

To analyze the data based on specialty, the IPM specialties were described as those providers designated in IPM -09, pain medicine -72, anesthesiology -05, physical medicine and rehabilitation -25, neurology -13, psychiatry -26, orthopedic surgery -20, and neurosurgery -14 [33]. General practitioners -01, family practitioners -08, and internists -11 were considered as general physicians. All other providers were considered as other physicians and providers.

### Data Synthesis

The data were analyzed using SPSS (9.0) statistical software, Microsoft Access 2003, and Microsoft Excel (2003). The procedure rates were calculated per 100,000 Medicare beneficiaries.

## Results

### Population Characteristics

Table 1 illustrates the characteristics of Medicare beneficiaries and facet joint interventions. During the same period, Medicare recipients receiving facet joint interventions increased 386%. Facet joint interventions increased from 606 per 100,000 in 1997 to 3,895 per 100,000 in 2006, a 543% increase.

**Table 1:** Characteristics of Medicare beneficiaries and facet joint interventions.

						% of increase from	
						
			1997	2002	2006	2002-2006	1997-2006
US Population (,000)			267,784	288,369	299,395	3.8%	11.8%

> = 65 years (,000)			34,933	35,602	37,125	4.3%	6.3%

Medicare Beneficiaries (,000)			38,465	40,503	43,339	7.0%	12.7%

Age		≥ 65 years	33,636	34,698	36,317	4.7%	8.0%
		
		< 65 years	4,829	5,805	7,022	21.0%	45.4%

Gender		Male	40.70%	43.85%	44.16%	0.7%	8.5%
		
		Female	59.30%	56.15%	55.84%	-0.6%	-5.8%

Facet joint intervention patients and visits							

Number of Medicare patients receiving facet joint interventions			46,640	119,160	254,720	114%	446%

Patients per 100,000 Medicare beneficiaries			121	294	588	100%	386%

Number of visits			88,280	225,280	543,900	141%	516%

Visits per 100,000 Medicare beneficiaries			230	556	1,255	126%	446%

Services			233,200	607,760	1,688,180	178%	624%

Interventions per 100,000 Medicare beneficiaries			606	1,501	3,895	160%	543%

Average visits per patient			1.9	1.9	2.1	0	0.2%

Facet joint interventions by age							

Patients	< 65 years	Number of patients	9,800	27,060	65,420	142%	568%
		
		Rate (per 100,000)	25	67	151	125%	504%
	
	≥ 65 years	Number of patients	36,840	92,100	189,300	106%	414%
		
		Rate (per 100,000)	96	227	437	93%	355%

Visits	< 65 years	Number of visits	19,840	54,960	154,760	182%	680%
		
		Rate (per 100,000)	52	136	357	163%	587%
	
	≥ 65 years	Number of visits	68,440	170,320	389,140	128%	469%
		
		Rate (per 100,000)	178	421	898	113%	404%

Services	< 65 years	Number of services	56,040	148,720	495,480	233%	784%
		
		Rate (per 100,000)	146	367	1,143	211%	683%
	
	≥ 65 years	Number of services	177,160	459,040	1,192,700	160%	573%
		
		Rate (per 100,000)	461	1,131	2,752	143%	498%

The results illustrate a higher proportion of increase for patients under 65; that proportion of patients increased 504% vs. 355%. For those 65 or over, visits increased 404% versus 587% for those under 65; services for those over 65 increased 498% versus 683% for those under 65. The Medicare population below the age of 65 years increased 45.4% in contrast to 8% of those 65 years or older.

### Utilization Characteristics

Table 2 illustrates the summary of frequency of utilization of facet joint interventions based on CPT code and place of service. Due to the 1997 data being non-comparable and not comprehensive, the data from 2002 and 2006 were utilized. The majority of the procedures (80% in 2002 and 77% in 2006) were performed in the lumbar region, with cervical and thoracic procedures constituting 20% in 2002 and 23% in 2006. The most commonly performed procedure was subsequent lumbar facet joint injection/nerve block (CPT 64476). Cervical/thoracic interventions increased 194% per 100,000 Medicare beneficiaries, whereas lumbar procedures increased 151%. In 2002, 40% of procedures were performed in HOPD settings and 41.7% in office settings; whereas in 2006, 59.6% were performed in office settings. The overall rate (per 100,000 Medicare beneficiaries) increased by 160% from 2002 to 2006; whereas in office settings the rate increased significantly (271%), followed by ASCs (168%) and HOPD settings (40%). Cervical procedures increased 194% with a distribution of 259%, 224%, and 59% in office, ASC, and HOPD settings.

**Table 2:** Utilization of facet joint interventions by place of service.

CPT	2002				2006				Change from 2002			
	
	Place of Service				Place of Service				Place of Service			
	
	ASC	HOPD	Office	Total	ASC	HOPD	Office	Total	ASC	HOPD	Office	Total
**Cervical/Thoracic (C/T)**												

64470	6,100	10,220	26,320	42,640	18,520	17,300	89,300	125,120	204%	69%	239%	193%

64472	10,380	19,380	34,360	64,120	34,340	32,300	145,400	212,040	231%	67%	323%	231%

***64470-72***	***16,480***	***29,600***	***60,680***	***106,760***	***52,860***	***49,600***	***234,700***	***337,160***	***221%***	***68%***	***287%***	***216%***

***Rate***	***41***	***73***	***150***	***264***	***122***	***114***	***542***	***778***	***200%***	***57%***	***261%***	***195%***

64626	1,020	2,280	1,400	4,700	4,700	3,580	5,340	13,620	361%	57%	281%	190%

64627	2,120	4,160	3,760	10,040	10,360	8,180	12,800	31,340	389%	97%	240%	212%

***64626-27***	***3,140***	***6,440***	***5,160***	***14,740***	***15,060***	***11,760***	***18,140***	***44,960***	***380%***	***83%***	***252%***	***205%***

***Rate***	***8***	***16***	***13***	***36***	***35***	***27***	***42***	***104***	***348%***	***71%***	***229%***	***185%***

												

**C/T Total**	**19,620**	**36,040**	**65,840**	**121,500**	**67,920**	**61,360**	**252,840**	**382,120**	**246%**	**70%**	**284%**	**215%**

**Rate**	**48**	**89**	**163**	**300**	**157**	**142**	**583**	**882**	**224%**	**59%**	**259%**	**194%**

**Lumbar/Sacral (L/S)**												

64475	26,120	60,340	69,960	156,420	67,580	84,420	214,160	366,160	159%	40%	206%	134%

64476	47,300	101,560	93,680	242,540	114,400	143,040	375,980	633,420	142%	41%	301%	161%

***64475-76***	***73,420***	***161,900***	***163,640***	***398,960***	***181,980***	***227,460***	***590,140***	***999,580***	***148%***	***40%***	***261%***	***151%***

***Rate***	***181***	***400***	***404***	***985***	***420***	***525***	***1,362***	***2,306***	***132%***	***31%***	***237%***	***134%***

64622	5,420	13,360	6,660	25,440	20,400	22,880	37,780	81,060	276%	71%	467%	219%

64623	12,660	31,660	17,540	61,860	47,940	51,840	125,640	225,420	279%	64%	616%	264%

***64622-23***	***18,080***	***45,020***	***24,200***	***87,300***	***68,340***	***74,720***	***163,420***	***306,480***	***278%***	***66%***	***575%***	***251%***

***Rate***	***45***	***111***	***60***	***216***	***158***	***172***	***377***	***707***	***253%***	***55%***	***531%***	***228%***

**L/S Total**	**91500**	**206,920**	**187,840**	**486,260**	**250,320**	**302,180**	**753,560**	**1,306,060**	**174%**	**46%**	**301%**	**169%**

**Rate**	**226**	**511**	**464**	**1,201**	**578**	**697**	**1,739**	**3,014**	**156**	**36%**	**275%**	**151%**

**Grand Total**												

**Services**	**111,120**	**242,960**	**253,680**	**607,760**	**318,240**	**363,540**	**1,006,400**	**1,688,180**	**186%**	**50%**	**297%**	**178%**

**Rate**	**274**	**600**	**626**	**1,501**	**734**	**839**	**2,322**	**3,895**	**168%**	**40%**	**271%**	**160%**

### Reimbursement Characteristics

Additional file 1 illustrates physician and facility reimbursement by place of service adjusted for inflation for years 2002 and 2006. As seen in Additional file 1, overall facility average charges decreased by 26%.

### Specialty Characteristics

**Figure 1:**
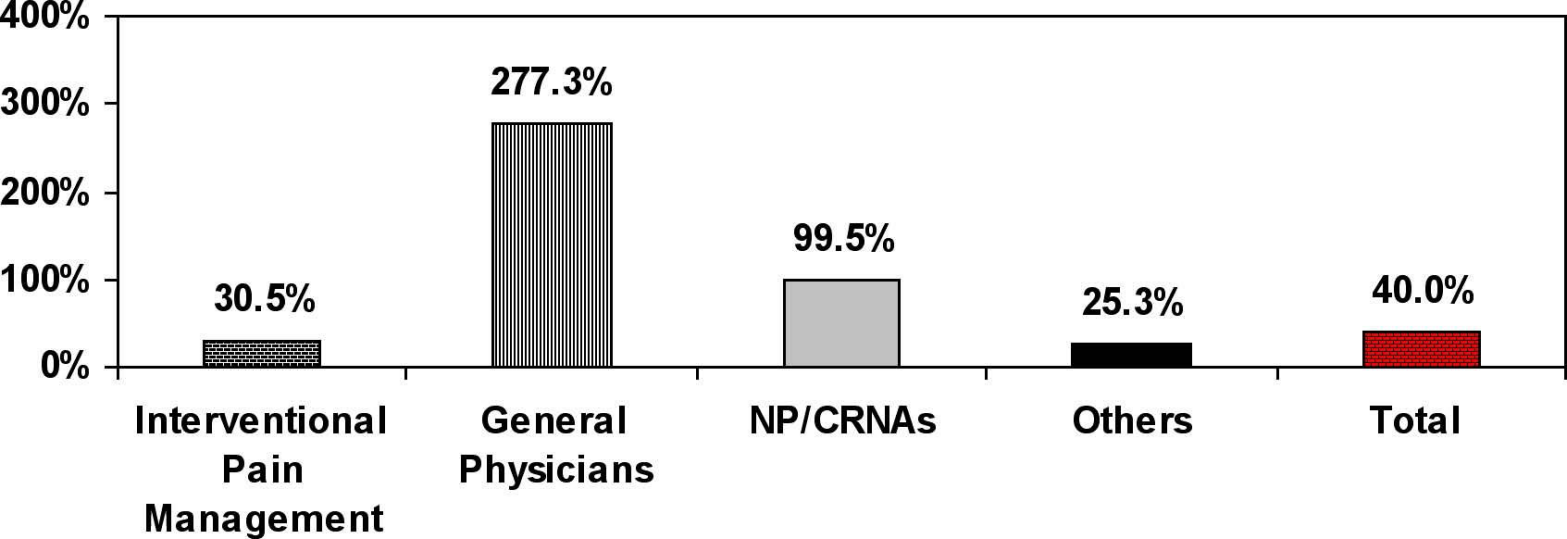
**Annual percentage of increase of facet joint intervention services per 100,000 Medicare recipients from 2002 to 2006**.

Figure 1 illustrates the increase in utilization of facet joint interventions by various specialty groups assigned as IPM, general practice, NPs/CRNAs, and others from 2002 to 2006. Across the country, the majority of procedures were performed by IPM physicians with 87% in 2002 and 74.5% in 2006. However, in 2006 general physicians performed 18.6% of these procedures, while all others performed 6.9% of the procedures (Table 3). Overall increases were greatest for general physicians, increasing by over 1,109% from 2002 to 2006, an annual growth of 277.3%. There was also an increase of 398% from 2002 to 2006 among NPs and CRNAs, an annual increase of 99.5%. In Florida in 2006, 47% of the procedures were performed by general physicians with specialties of general practice, family practice, and internal medicine.

**Table 3:** Utilization of facet joint interventions by speciality.

	2002			2006			Change from 2002	
**Speciality**	**Services**	**Percent**	**Rate**	**Services**	**Percent**	**Rate**	**Percent**	**Rate**

**Interventional Pain Management**	**529,220**	**87.1%**	**1,307**	**1,256,860**	**74.5%**	**2,900**	**-15%**	**122%**

Anesthesiology	338,660	55.7%	836	524,340	31.1%	1,210	-44%	45%

Pain Management	78,080	12.8%	193	459,520	27.2%	1,060	112%	450%

***Anesthesiology & Pain Management***	***416,740***	***68.5%***	***1,029***	***983,860***	***58.3%***	***2,270***	***-15%***	***121%***

Physical Medicine and Rehabilitation	54,000	8.9%	133	148,980	8.8%	344	-1%	158%

Orthopedic Surgery	24,600	4.0%	61	51,860	3.1%	120	-24%	97%

Neurology	23,140	3.8%	57	49,400	2.9%	114	-23%	100%

Neurosurgery	9,320	1.5%	23	21,080	1.2%	49	-19%	111%

Psychiatry	1,420	0.2%	4	1,680	0.1%	4	-57%	11%

**Family & General Practice/Internal Medicine**	**24,300**	**4.0%**	**60**	**314,420**	**18.6%**	**725**	**366%**	**1109%**

**Others**	**54,240**	**8.9%**	**134**	**116,900**	**6.9%**	**270**	**-22%**	**101%**

Diagnostic Radiology	14,100	2.3%	35	20,140	1.2%	46	-49%	33%

Nurse Practitioners/CRNA's	860	0.1%	2	4,580	0.3%	11	92%	398%

Others	39,280	6.5%	97	92,180	5.5%	213	-16%	119%

								

**Total**	**607,760**	**100%**	**1,501**	**1,688,180**	**100%**	**3,895**	**178%**	**160%**

### Fluoroscopy Utilization

**Figure 2:**
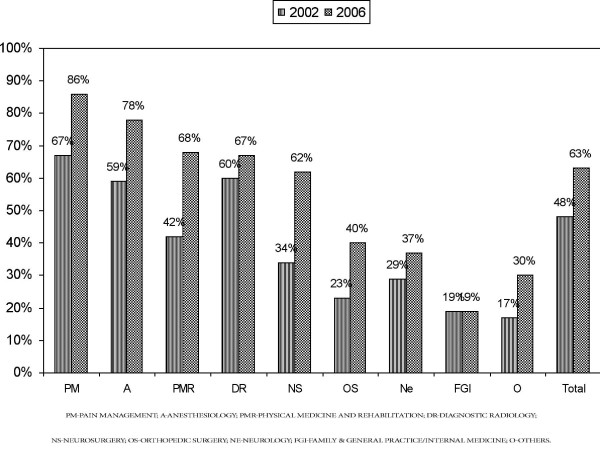
**Percentage of visits utilizing fluoroscopy based on specialty**.

Figure 2 illustrates fluoroscopy utilization based on specialty. Overall in 2002, 48% of all visits included fluoroscopy, compared to 63% visits of all visits in 2006.

### Procedural Characteristics by State

Table 4 illustrates facet joint interventions for each state. South Dakota showed the highest increase of 504% with Alabama showing the lowest increase of 14% per 100,000 Medicare beneficiaries. The overall increase for the United States was 160% from 2002 to 2006. However, smaller states with a small number of procedures, such as South Dakota, preclude any conclusions to be drawn as per the increases. Thus, when normalized for population, Florida showed a 26.8-fold difference from Hawaii, the state with the lowest, for 2006. All other states showed a difference of less than 10-fold with Michigan showing a 9.87-fold difference, Texas showing an 8.42-fold difference, Arkansas showing a 7.34-fold difference, and Delaware showing a 6.47-fold difference, compared to the lowest state for 2006. Further, facet joint procedures per state as a proportion of national utilization declined in multiple states.

**Table 4:** Number of facet joint interventions and procedures per 100,000 Medicare beneficiaries provided by state.

	2002		2006		% of change from 2002		Fold difference from the lowest state for 2006
	
State	Services	Rate per 100,000 population	Services	Rate per 100,000 population	Services	Rate per 100,000 population	
Florida	108,800	3,603	534,000	17,340	391%	381%	26.80

Michigan	44,940	3,514	96,460	6,386	115%	82%	9.87

Texas	62,680	2,680	142,960	5,445	128%	103%	8.42

Arkansas	8,240	1,692	23,040	4,752	180%	181%	7.34

Delaware	800	714	5,520	4,187	590%	486%	6.47

Alaska	400	874	2,000	4,026	400%	361%	6.22

Mississippi	6,920	1,788	16,600	3,596	140%	101%	5.56

Kentucky	11,520	1,797	24,900	3,583	116%	99%	5.54

Utah	2,620	1,365	8,440	3,431	222%	151%	5.30

Tennessee	12,440	1,695	32,460	3,419	161%	102%	5.29

West Virginia	3,160	878	12,080	3,343	282%	281%	5.17

Montana	2,740	1,745	5,060	3,335	85%	91%	5.15

Maryland	8,500	1,302	23,320	3,294	174%	153%	5.09

North Carolina	15,840	1,331	42,400	3,218	168%	142%	4.97

Ohio	17,620	1,134	56,060	3,153	218%	178%	4.87

Vermont	800	875	2,900	3,150	263%	260%	4.87

South Carolina	6,540	965	21,160	3,140	224%	225%	4.85

Missouri	8,260	1,109	29,160	3,137	253%	183%	4.85

New Hampshire	3,320	2,024	6,200	3,134	87%	55%	4.84

Alabama	20,220	2,682	23,620	3,058	17%	14%	4.73

Indiana	12,620	1,485	28,140	3,050	123%	105%	4.71

Pennsylvania	31,560	1,552	63,740	2,957	102%	90%	4.57

Georgia	14,820	1,705	31,360	2,916	112%	71%	4.51

South Dakota	580	480	3,460	2,904	497%	504%	4.49

Iowa	7,780	1,784	13,960	2,823	79%	58%	4.36

Louisiana	4,220	701	17,500	2,804	315%	300%	4.33

Arizona	5,960	753	22,540	2,765	278%	267%	4.27

Wyoming	780	1,158	1,780	2,593	128%	124%	4.01

Massachusetts	10,280	1,155	25,240	2,571	146%	123%	3.97

California	55,060	1,458	103,000	2,409	87%	65%	3.72

Wisconsin	10,060	1,435	19,660	2,341	95%	63%	3.62

Maine	2,640	1,153	5,560	2,311	111%	100%	3.57

New York	27,660	1,057	63,840	2,276	131%	115%	3.52

New Mexico	2,720	925	6,120	2,219	125%	140%	3.43

Kansas	2,000	531	8,980	2,209	349%	316%	3.41

Illinois	17,060	1,054	37,180	2,171	118%	106%	3.35

Nevada	2,640	996	6,580	2,145	149%	115%	3.32

Virginia	10,720	1,203	19,900	1,955	86%	62%	3.02

New Jersey	13,320	1,073	23,180	1,867	74%	74%	2.89

Colorado	4,740	946	10,020	1,856	111%	96%	2.87

Oklahoma	5,920	1,159	10,260	1,854	73%	60%	2.86

Connecticut	3,040	559	9,160	1,728	201%	209%	2.67

Minnesota	3,440	587	11,940	1,674	247%	185%	2.59

Idaho	1,760	1,019	3,100	1,656	76%	63%	2.56

Nebraska	1,100	430	3,440	1,382	213%	222%	2.14

Washington	4,560	667	11,560	1,365	154%	105%	2.11

Rhode Island	880	511	2,060	1,332	134%	161%	2.06

Oregon	1,440	295	7,240	1,310	403%	344%	2.02

North Dakota	960	930	1,160	1,184	21%	27%	1.83

District of Columbia	360	485	620	1,021	72%	110%	1.58

Hawaii	720	420	1,100	647	53%	54%	1.00

Overall	607,760	1,501	1,688,180	3,895	178%	160%	6.02

### Diagnostic Characteristics

Table 5 illustrates the utilization of ICD-9-CM diagnostic codes for facet joint interventions. The most common diagnoses documented were "lumbosacral spondylosis" in the lumbar spine of 32.3% and cervical spondylosis in the cervical spine of 5.3%. Degenerative disc disease was the diagnosis criteria utilized in 6.2% and 1.2% of cases in the lumbar and cervical spine respectively. Thus, accurate diagnosis was utilized in fewer than 50% of patients in 2006.

**Table 5:** Line of diagnosis for facet joint interventions.

Group	2002	Percent	2006	Percent
LUMBOSACRAL SPONDYLOSIS	168,980	32.3%	3,379,600	32.3%
LUMBAGO/BACK PAIN	151,240	28.9%	3,024,800	28.9%
CERVICAL SPONDYLOSIS W/WO MYELOPATHY	27,960	5.3%	559,200	5.3%
DEGENERATION OF LUMBAR OR LUMBOSACRAL INTERVERTEBRAL DISC	32,180	6.2%	643,600	6.2%
CERVICALGIA	29,320	5.6%	586,400	5.6%
SCIATICA	2,800	0.5%	56,000	0.5%
THORACIC OR LUMBOSACRAL NEURITIS OR RADICULITIS UNSPECIFIED	21,680	4.1%	433,600	4.1%
THORACIC SPONDYLOSIS W/WO MYELOPATHY	4,320	0.8%	86,400	0.8%
SPINAL STENOSIS	11,940	2.3%	238,800	2.3%
POSTLAMINECTOMY SYNDROME	10,860	2.1%	217,200	2.1%
DEGENERATION OF CERVICAL INTERVERTEBRAL DISC	6,040	1.2%	120,800	1.2%
LUMBAR DISC DISPLACEMENT	6,980	1.3%	139,600	1.3%
PAIN IN JOINT UNSPECIFIED/SPECIFIED AREA	5,320	1.0%	106,400	1.0%
BRACHIAL NEURITIS OR RADICULITIS NOT OTHERWISE SPECIFIED	4,560	0.9%	91,200	0.9%
ARTHROPATHY	1,680	0.3%	33,600	0.3%
OTHER SYNDROMES AFFECTING CERVICAL REGION	5,640	1.1%	112,800	1.1%
POSTLAMINECTOMY SYNDROME OF CERVICAL REGION	1,000	0.2%	20,000	0.2%
LUMBOSACRAL SPRAIN	1,680	0.3%	33,600	0.3%
DEGENERATION OF THORACIC OR THORACOLUMBAR INTERVERTEBRAL DISC	1,220	0.2%	24,400	0.2%
CONGENITAL ANOMALIES OF SPINE	1,200	0.2%	24,000	0.2%
DISORDERS OF SACRUM	2,300	0.4%	46,000	0.4%
SPONDYLOLISTHESIS	1,120	0.2%	22,400	0.2%
MYALGIA AND MYOSITIS	1,560	0.3%	31,200	0.3%
DEGENERATION OF INTERVERTEBRAL DISC SITE UNSPECIFIED	940	0.2%	18,800	0.2%
NEURALGIA NEURITIS AND RADICULITIS UNSPECIFIED	520	0.1%	10,400	0.1%
OSTEOARTHROSIS	1,180	0.2%	23,600	0.2%
SPINAL STENOSIS IN CERVICAL REGION	800	0.2%	16,000	0.2%
SPASM OF MUSCLE	840	0.2%	16,800	0.2%
PATHOLOGICAL FRACTURE OF VERTEBRAE	400	0.1%	8,000	0.1%
LUMBOSACRAL PLEXUS LESIONS	800	0.2%	16,000	0.2%
INFLAMMATORY SPONDYLOPATHY	520	0.1%	10,400	0.1%
OTHERS	12,840	2.5%	256,800	2.5%

## Overall Growth Pattern

**Figure 3:**
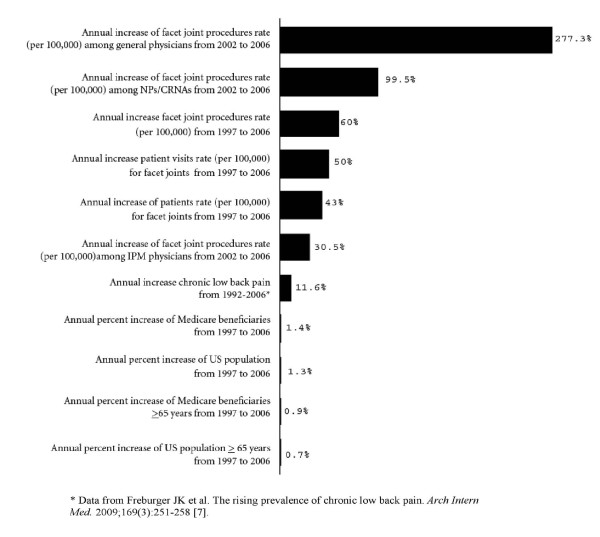
**Illustration of overall annual growth patterns**.

Figure 3 illustrates the overall growth pattern of facet joint interventions. These annual rates of increase for facet joint interventions represent the years from 1997 to 2006. There was an increase of facet joint interventions by general physicians of over 1,109%.

## Discussion

Facet joint intervention rates for spinal disorders increased dramatically over the study period from 1997 to 2006. This increase per 100,000 Medicare population from 1997 to 2006 was relatively constant over time, resulting in an increase of facet joint patients of 386%, facet joint visits of 446%, and facet joint interventions of 543%. Facet joint interventions also increased based on age. Among Medicare recipients per 100,000, less than 65 years of age, compared to those 65 or older, the patient population receiving facet joint interventions increased 504% vs. 355%, visits increased 587% compared to 404%, and services increased 683% compared to 498%. In addition, total expenditures also increased from over $229 million in 2002 to over $511 million in 2006, with an overall increase of 123% from 2002 to 2006. There was a significant increase of 1,109% in the utilization of facet joint interventions by general physicians -- composed of general practice, family practice, and internal medicine -- from 2002 to 2006, an annual increase of 277.3%. There were also significant usage or utilization increases among NPs and CRNAs from 2002 to 2006 of 398%, an annual increase of 99.5%. These increases were substantially higher than any other specialty, even though overall increases were significant: 160% from 2002 to 2006, an annual increase of 40%.

There was a 26.8-fold difference in the utilization pattern in Florida from Hawaii, the state with the lowest pattern for 2006. The remaining 49 states showed less than a 10-fold difference. Further, it has been shown that 47% of facet joint interventions in Florida were performed by general physicians. There has been an exponential growth of facet joint interventions in office settings of 271% with ASC settings showing 168% growth and HOPD settings showing 40% growth. However, moving the procedures to hospital settings will not resolve the issue as the average cost of the total procedure in HOPD settings in 2006 was $467.80, whereas in in-office settings, it was $227.60 and in ASC settings, it was $352.20.

Fluoroscopy utilization was lowest among family and general practice and internal medicine physicians and highest among pain management specialties. Non-fluoroscopically guided procedures present multiple issues regarding the accuracy of the procedure, medical necessity, and documentation.

With respect to evidence for facet joint interventions, there is emerging evidence to show the effectiveness of medial branch blocks and radiofrequency neurotomy along with effective diagnosis, when patients are selected appropriately meeting indications and medical necessity criteria [20-28]. While this evidence is emerging, some systematic reviews [19] have not utilized these trials [26-28] in their evidence synthesis.

Friedly et al [3] postulated that there was a disproportionate increase in procedures in ACSs, and that ACSs received higher payments. The implication is that these procedures had been shifted to ACSs as self referrals. Also that there was excessive use by facilitating physician investors to increase practice revenues by receiving facility payments for procedures. However, our study shows that this is not an issue. Rather, it may be due to the providing of more efficient services as a result of specialized staff and equipment, and convenient locations with short waiting times as well as better physician production. Further, the data illustrates that the procedures are more expensive in HOPD settings compared to ASC settings.

Based on the current data, it appears that the annual increase in the population with chronic low back pain is 11.6% [7], and the increase in facet joint intervention visits is approximately 50%. The increases are much lower in states with stricter regulations and LCDs [34,35]. Kentucky showed an annual increase of 25% and Indiana, 26%; whereas the annual increase in Florida was 95%. The overall increase across the country was 40% from 2002 to 2006.

McKinsey Global Institute [30] postulated multiple factors for the increased growth of outpatient health care services in the United States. First, provider capacity growth and response to high outpatient margins is illustrated in this study based on significant increases in in-office settings and also performing these procedures. Other causes are that in outpatient settings, more efficient services are provided as a result of specialized staff and equipment, convenience of the location, short waiting times, and better physician production [34,35]. The second factor relates to judgment based on the nature of physician care. Over the years there has been significant growth in interventional pain management due to increased understanding and to the availability of a supply of physicians. The third factor described relates to technological innovation that drives prices higher rather than lower [36], which is not proven in this study in the Medicare population in the United States. The fourth factor relates to demand growth that appears to be due to the greater availability of supplies. While this is accurate, there is also demand due to access and also to the increasing prevalence of spinal pain. The final factor relates to relatively price-insensitive patients with limited out-of-pocket costs. This factor may be realistic in the overall health care evaluation. However, in the Medicare population, the application of this is minimal. In this study we included only the patients who were paying fee-for-service. Thus, price insensitivity does not apply. However, the study of the patients with third party insurance with low out-of-pocket costs and workers' compensation patients with no out-of-pocket costs and Medicare Advantage patients with low out-of-pocket costs or no out-of-pocket costs will illustrate these differences. Yet numerous problems continue to exist with overuse and abuse.

There are multiple limitations to our study. These include the lack of inclusion of participants in Medicare Advantage plans, which includes approximately 10% of enrollees, and potential coding errors [3,31]. However, we have included all patients over 65 receiving traditional fee-for-service Medicare and under 65 as well. This inclusion is important because patients below the age of 65 represent a significant proportion of patients receiving facet joint interventions, with a higher frequency of services. In general, patients less than 65 years of age received more intense and a higher proportion of services (504% vs. 355%) [2]. This fact is echoed in this evaluation, which shows an increase of facet joint services of 683% vs. 498% from 1997 to 2006. Since the data does not contain HOPD facility charges, we had to estimate the facility charges for outpatient hospital charges, similar to Friedly et al [3]. Another limitation is that some variation may be related to coding errors and diagnostic ambiguity, and to non-reporting of fluoroscopy. However, due to the usage of actual data for physicians, ASCs, and office services, these errors should have very little influence.

Multiple recommendations have been made to slow the growth of health care costs in general and for interventional techniques in particular [1,4,36]. Health care experts have recommended policies that encourage high-growth or high-cost regions to behave more like slow-growth, low-cost regions and to encourage low-cost, slow-growth regions to sustain their current needs for interventional techniques to slow spending growth. The OIG [1] has recommended strengthening program efforts to prevent improper payments; others [3] have also recommended more stringent regulations on medical necessity, indications, accreditation provisions in the settings performed, and training and qualifications of the physicians performing the procedures.

## Conclusion

In conclusion, our data summarizes the explosive growth of facet joint interventions in agreement with the OIG report [1] and other reports [2]. This review also demonstrates that the growth has been substantial in certain regions and by certain specialties. Some of the growth may be accounted for by improved access, precision of diagnostic and therapeutic modalities outcomes, and the increasing prevalence of spinal pain. However, there still continue to be multiple problems with ambiguity of diagnosis, lack of fluoroscopic use, disproportionate increase in procedures by some specialties and some regions, and escalating costs.

## Competing interests

Dr. Manchikant is CEO and Chairman of American Society of Interventional Pain Physician representing interventional pain physicians across the nation; is Medical Director of the Pain Management Center of Paducah, the Ambulatory Surgery Center of Paducah, and Pain Care Surgery, providing interventional pain management services including facet joint interventions in an ambulatory surgery center and in an office setting; and is Associate Professor of Anesthesiology at The University of Louisville.

Mr. Pampati is a statistician and employed by Pain Management Center of Paducah. He is a non-physician and does not performing interventional techniques; however, he is involved extensively in statistical management of data related to interventional pain management.

Dr. Singh is Medical Director of the Pain Diagnostic Associates, Niagara, Wisconsin. He is an interventional pain management physician practicing in an ambulatory surgery center and in hospital outpatient departments including facet joint interventions.

Dr. Boswell is a Professor of Anesthesiology and Director of the International Pain Center, Texas Tech University Health Sciences Center, Lubbock, Texas. He practicing interventional pain management including Interventional techniques in all 3 settings; in hospital, hospital outpatient, and in-office settings.

Dr. Smith is an Associate Professor and Academic Director of Pain Management for Albany Medical College Department of Anesthesiology, Albany, New York. He practices interventional pain management including interventional techniques in hospital outpatient department setting.

**Dr. Hirsch **is Chief of Minimally Invasive Spine Surgery with the Departments of Radiology and Neurosurgery, Massachusetts General Hospital; and an Associate Professor of Radiology, Harvard Medical School, Boston, Massachusetts. His practice also includes interventional radiology including interventional procedures, but does not include facet joint interventions.

## Authors' contributions

LM, VP, VS, MVB, HSS, and JAH conceived the concept, design and coordination. VP processed the data. LM, VS, and MVB drafted the manuscript. HSS and JAH participated in the revision of the manuscript. All authors read and approved the final manuscript.

## Pre-publication history

The pre-publication history for this paper can be accessed here:

http://www.biomedcentral.com/1472-6963/10/84/prepub

## Supplementary Material

Additional file 1 Summary of the frequency of utilizations of various facet joint interventions in Medicare beneficiaries based on place of service in 2002 and 2006. Click here for file
