# Supplementary material for: Explosive growth of facet joint interventions in the medicare population in the United States: a comparative evaluation of 1997, 2002, and 2006 data
Source: BMC Health Serv Res. 2010 Mar 30;10:84. doi: 10.1186/1472-6963-10-84 (PMC2907752; doi:10.1186/1472-6963-10-84)
Supplement: Additional file 1 — Summary of the frequency of utilizations of various facet joint interventions in Medicare beneficiaries based on place of service in 2002 and 2006. [file 1472-6963-10-84-S1.DOC]

**Table 3.** *Summary of the frequency of utilization and reimbursement characteristics of facet joint interventions in Medicare beneficiaries based on place of service in 2002 and 2006.*

|  | **2002** | | | | | **2006** | | | | | **Change from 2002** | |
| --- | --- | --- | --- | --- | --- | --- | --- | --- | --- | --- | --- | --- |
| **Physician** | **Patients** | **Visits** | **Services** | **Total Charges** | **Average**  **Per Service** | **Patients** | **Visits** | **Services** | **Total Charges** | **Average**  **Per Service** | **Total Charges** | **Average Charges** |
| ASC | 20,420 | 38,440 | 111,120 | $ 9,280,366 | $83.5 | 55,240 | 104,860 | 318,240 | $28,636,140 | $90.0 | 209% | 8% |
| HOPD | 50,880 | 85,020 | 242,960 | $ 19,525,511 | $80.4 | 69,660 | 121,660 | 363,540 | $30,992,900 | $85.3 | 59% | 6% |
| Office | 47,860 | 101,820 | 253,680 | $19,468,909 | $76.7 | 129,820 | 317,380 | 1,006,400 | $ 77,711,663 | $77.2 | 299% | 1% |
| Total | 119,160 | 225,280 | 607,760 | $ 48,274,786 | $79.4 | 254,720 | 543,900 | 1,688,180 | $137,340,703 | $81.4 | 184% | 2% |
| **Facility** |  |  |  |  |  |  |  |  |  |  |  |  |
| ASC | 20,420 | 38,440 | 111,120 | $ 32,518,364 | $292.6 | 55,240 | 104,860 | 318,240 | $83,451,660 | $262.2 | 157% | -10% |
| HOPD | 50,880 | 85,020 | 242,960 | $108,697,109 | $447.4 | 69,660 | 121,660 | 363,540 | $139,071,100 | $382.5 | 28% | -14% |
| Office | 47,860 | 101,820 | 253,680 | $39,551,815 | $155.9 | 129,820 | 317,380 | 1,006,400 | $151,316,037 | $150.4 | 283% | -4% |
| Total | 119,160 | 225,280 | 607,760 | $180,767,288 | $297.4 | 254,720 | 543,900 | 1,688,180 | $373,838,797 | $221.4 | 107% | -26% |
| **Total** |  |  |  |  |  |  |  |  |  |  |  |  |
| ASC | 20,420 | 38,440 | 111,120 | $41,798,730 | $376.2 | 55,240 | 104,860 | 318,240 | $112,087,800 | $352.2 | 168% | -6% |
| HOPD | 50,880 | 85,020 | 242,960 | $128,222,620 | $527.8 | 69,660 | 121,660 | 363,540 | $170,064,000 | $467.8 | 33% | -11% |
| Office | 47,860 | 101,820 | 253,680 | $59,020,724 | $232.7 | 129,820 | 317,380 | 1,006,400 | $229,027,700 | $227.6 | 288% | -2% |
| Total | 119,160 | 225,280 | 607,760 | $229,042,074 | $376.9 | 254,720 | 543,900 | 1,688,180 | $511,179,500 | $302.8 | 123% | -20% |
